# Cultural adaptation and validation of the caring behaviors assessment tool into Spanish

**DOI:** 10.1186/s12912-024-01892-2

**Published:** 2024-04-10

**Authors:** Juan M. Leyva-Moral, Carolina Watson, Nina Granel, Cecilia Raij-Johansen, Ricardo A. Ayala

**Affiliations:** 1 Nursing Department, Faculty of Medicine, Universitat Autònoma de Barcelona, Av. Can Domènech S/N, Cerdanyola del Vallès, 08193 Spain; 2 Universidad de Las Américas, Santiago de Chile, Chile; 3 Ghent University, Ghent, Belgium

**Keywords:** Caring behaviors, Transculturation, Validation, Humanization, Nursing care

## Abstract

**Background:**

The aim of the research was to translate, culturally adapt and validate the Caring Behaviors Assessment (CBA) tool in Spain, ensuring its appropriateness in the Spanish cultural context.

**Methods:**

Three-phase cross-cultural adaptation and validation study. Phase 1 involved the transculturation process, which included translation of the CBA tool from English to Spanish, back-translation, and refinement of the translated tool based on pilot testing and linguistic and cultural adjustments. Phase 2 involved training research assistants to ensure standardized administration of the instrument. Phase 3 involved administering the transculturally-adapted tool to a non-probabilistic sample of 402 adults who had been hospitalized within the previous 6 months. Statistical analyses were conducted to assess the consistency of the item-scale, demographic differences, validity of the tool, and the importance of various caring behaviors within the Spanish cultural context. R statistical software version 4.3.3 and psych package version 2.4.1 were used for statistical analyses.

**Results:**

The overall internal consistency of the CBA tool was high, indicating its reliability for assessing caring behaviors. The subscales within the instrument also demonstrated high internal consistency. Descriptive analysis revealed that Spanish participants prioritized technical and cognitive aspects of care over emotional and existential dimensions.

**Conclusions:**

The new version of the tool proved to be valid, reliable and culturally situated, which will facilitate the provision of objective and reliable data on patients beliefs about what is essential in terms of care behaviors in Spain.

**Supplementary Information:**

The online version contains supplementary material available at 10.1186/s12912-024-01892-2.

## Key points

• This paper provides a culturally translated, adapted, and validated version of the Caring Behaviors Assessment tool in the Spanish context, which can be used to obtain reliable and culturally adapted data on essential aspects of patient care.

• The findings of this study contribute to the wider global clinical community by demonstrating the importance of considering cultural factors when assessing and evaluating patient care from patients’ own perspective, and also emphasizes the need for culturally sensitive approaches in healthcare settings.

• This validated instrument facilitates the measurement of caring behaviors in the Spanish context, allowing for objective evaluation and improvement. Use of the Caring Behaviors Assessment tool could thus serve as a valuable resource for both future research and clinical practice.

**Supplementary Information:**

The online version contains supplementary material available at 10.1186/s12912-024-01892-2.

## Background

*Caring,* as a complex culturally derived phenomenon, encompasses recognition of individuals’ uniqueness and includes moral, emotional, and cognitive dimensions [1]. Within the field of nursing, the professional act of caring is defined as an interpersonal process characterized by nurses’ expertise, competencies, personal maturity, and interpersonal sensitivity. The ultimate aim is to meet patients’ bio-psycho-social needs, ensuring their protection, emotional support, and overall satisfaction [2]. Furthermore, caring has been understood as the pivotal element that patients expect and should encounter to feel satisfied with nursing services [3]. Therefore, the concept of caring is dynamic, requiring adaptation to diverse sociocultural contexts.

Drawing on humanistic, transformative, integrative, and complex ontological and epistemological perspectives, various nursing theories have been developed that focus on promoting human-centred care [4, 5]. One such perspective is the theory of human-to-human relationships proposed by Travelbee [6], which emphasizes the unique and irreplaceable nature of anyone who has lived or will live in this world. In this perspective, therapeutic human relationships evolve through a series of interactive steps, including the emergence of identities and the development of empathy (and later sympathy) until finally establishing rapport with persons receiving care [7].

Similarly, Watson [8, 9] has elaborated a care process consisting of the following ten steps (caritas process): 1) consciously practising kindness and honesty while providing care; 2) being authentically present in a facilitative manner; 3) cultivating spirituality by transcending the self; 4) developing and maintaining a relationship of trust; 5) supporting the expression of both positive and negative feelings; 6) using creativity to obtain information during the care process; 7) engaging in genuine teaching and learning that take a global view of phenomena, while considering the perspective of the other; 8) creating healing environments that enhance integrity, comfort, dignity, and peace; 9) consciously and intentionally assisting with basic needs while enhancing the mind, body, and spirit; 10) remaining open to the experience of life and death, including care of both the professional and the patient’s soul. In short, caring is the essence of nursing and is a fundamental element for establishing effective nurse-patient relationships and achieving high-quality health outcomes.

The quality of nursing care is directly related to patients’ general experience and satisfaction. Evidence shows that patient experience with nursing care is a crucial predictor of patient satisfaction [10, 11]. Studies indicate that providing expert and integrated care contributes to patients’ sense of safety and feeling embraced [12]. Conversely, professional nursing practice based on the biomedical model has been associated with low patient satisfaction and limited professional fulfilment among nurses [13].

Nevertheless, measuring nursing care plays an essential role in assessing its effectiveness and quality. By measuring nursing care, healthcare organisations and policymakers can identify areas for improvement and make evidence-based decisions to enhance patient outcomes. While caring cannot be reduced to a mere collection of actions and behaviours, this step is crucial in systematising the components of care that impact patients’ experiences [14] and in determining the contribution of nursing to health systems [15]. Watson [9] argues that, without engaging in philosophical contradictions, the use of quantitative instruments to assess care is necessary to provide scientific evidence. Such evidence helps managers and researchers to evaluate the complex and unique role of nursing and its effects on health.

The presence of an adequate number of well-trained nurses is known to reduce the risk of patient mortality, with outcomes similar to those achieved by physicians [16]. Nevertheless, nursing care extends beyond numerical values and clinical outcomes. It is well-established that discrepancies exist between the perceptions of nurses and patients regarding what constitutes care, primarily due to the uniqueness of each individual; hence the application of individualized care is promoted and takes into account the sociocultural context [17]. Moreover, humanised care is associated with high levels of patient and family satisfaction in various contexts [18].

One of the oldest and most widely used tools for assessing nursing care is the Caring Behaviours Assessment (CBA) tool, developed by Cronin and Harrison [19]. The authors were concerned about the exclusion of patients’ perspective in care settings and sought to identify which behaviours communicated care and how their effectiveness could be evaluated. Consequently, they created and validated the CBA, which comprises 63 items, grouped into seven subscales based on Watson’s ten carative factors. The instrument has been translated and validated in several languages, including Chilean Spanish [15]. However, the Spanish spoken in Spain exhibits distinct differences to the Chilean variety in word usage, meaning and cultural nuances, influenced by other languages spoken in the country such as Catalan or Galician. Consequently, despite extensive debate in recent years, there are currently no reliable assessment instruments available in the Spanish context that adequately consider cultural nuances in patients’ experiences. Therefore, using the CBA in an apparently similar but different language variety could lead to misinterpretation [20].

## Methods

The aim of this study is to report the process of cultural translation, adaptation, and validation of the CBA in Spain, which to the best of our knowledge is the only culturally grounded version available. This new version of the CBA will provide a reliable means to obtain objective, tangible, and culturally adapted data on patients’ perceptions of the elements they deem to be essential in their care.

Approval was obtained from the relevant Ethics Committee on 2020 (*ethics committee name, hidden for blinding purposes*). Then, a study organised in three phases was undertaken on 2021–2022. The phases were as follows: 1. Transculturation. 2. Training. 3. Administration.

### Phase 1

A previous publication reported the process of creating a version of the CBA in Latin-American Spanish, namely in Chile. The authors of that publication suggested several steps for obtaining a transculturally adapted version, which we used here. These steps were as follows:

- *Translating the CBA from English to Spanish*: one translation (draft 1) was done by a non-nursing translator, and another one (draft 2) by two bilingual nurses, who were familiar with Watson’s theory. The two drafts were then contrasted, leading to an agreed translation (draft 3).
- *Back-translation from Spanish into English*: A bilingual nurse who was familiar with the subject but unfamiliar with the CBA, back-translated draft 3 into English (draft 4).
- *Refining the Spanish draft prior to the pilot test*: the authors reworked a refined version (draft 5) by contrasting the back-translation with the original CBA in English.
- *Pilot-testing the translated version*: Once satisfactorily refined, the translated version was tested with 36 volunteers. This step included interviewing them to identify their understanding of each item.
- *Linguistic and cultural adjustment:* draft 5 was further adjusted by analyzing the volunteers’ responses and using three linguistic criteria: semantic disambiguation, morpho-syntax, and language. This step aimed to ensure one of the key traits of the CBA: plain language. As in the Latin-American version by Ayala and Calvo [15], conjugation was adjusted (i.e., use of the subjunctive tense instead of the present tense), so that the items reflected hypothetical situations. Otherwise, it would be all too easy for patients to misconstrue that they were being asked to assess the actual care provided by specific nursing staff. Equally, the order of the Likert-type scale was maintained from 1 to 5, left to right. Lastly, grammatical structures and words that sounded natural in spoken Spanish were double-checked with a linguistic consultant. This process led to the preliminary version of the CBA in Spanish.

### Phase 2

A team of research assistants was trained in the application of the instrument to ensure a standardised administration process. The training included, for example, that informed consent had to be obtained from all participants before they were given a copy of the questionnaire, that the instructions had to be read aloud to the participants clearly and calmly, that the instrument had to be completed privately, and that the assistants had to remain nearby and attend to participants’ queries. This phase was crucial to minimize the risk of inducing an observer effect on responses.

### Phase 3

We administered the transculturally-adapted version of the CBA to a non-probability sample (*N* = 402). To test its psychometric properties [21], the preliminary version was applied to a sample of adults (between 5 and 10 per item; with a mean age of 39.5 years [SD = 16.5]), who had been hospitalised within the previous 6 months (mean = 2.75 times). This phase aimed to assess the CBA with users of similar characteristics and under similar conditions to those of the final intended users: the CBA is specifically designed to be used in hospital settings.

The procedure yielded 402 observations, providing a significant amount of data for the analysis of item/scale and subscale/scale consistency, as well as the overall reliability of the CBA in measuring a single construct. Of the 402 observations, 120 were excluded from the analysis as they were from health practitioners. As a result, the final sample size was for the analysis was *N* = 282.

#### Statistical analysis

Our objective was to analyse the single items and item‐scale consistency, as well as explain potential differences in perceptions based on demographic data. In addition to assessing the validity of the scale, we also aimed to determine the relevance of diverse caring behaviours within the particular cultural setting of the study. To achieve this, we used correlation analyses to examine the associations between caring behaviors and relevant cultural factors.

Analyses were performed by examining mean and SD (± 1SD) values per item to identify the highest‐ and the lowest‐ranking behaviours. In addition, a Kaiser–Meyer–Olkin (KMO) factor adequacy and Bartlett’s test for sphericity were used to know if our dataset could be factored. Afterwards, Exploratory Factor Analysis (EFA) was used to find common structure in data. The final number of factors was obtained using a parallel analysis. The factorial method employed was minimum residual with Varimax rotation.

Finally, Cronbach’s alpha as well as McDonald’s omega were used to estimate internal consistency and reliability respectively. All statistical analyses were performed using R statistical software (v4.3.3) [22] and the package psych (v2.4.1) [23].

## Results

As previously mentioned, 120 out of the 402 participants were health professionals. Our initial intention was to retain them in the sample, but their responses made the items markedly redundant, likely due to their familiarity with philosophies of care or a self-validating effect. Therefore, these participants were excluded from the sample. The paragraphs below report the results of the validation tests.

### Scores by items

As per descriptive statistics, we calculated mean scores ± 1SD for each of the 63 items of the CBA. The five highest‐ranking and five lowest‐ranking behaviours are listed below (Tables 1 and 2). The means ranged from a maximum of [4.87] (± 0.44) for item 3 “Know what they’re doing” to a minimum of [2.88] (± 1.06) for item 25 “Visit me if I move to another hospital unit.”

**Table 1:** Mean and standard deviation (± 1SD) for the five highest-ranking items

**Item**	**Mean (**± 1**SD)**
16. Treat me with respect	4.78 (± 0.53)
60. Know when it’s necessary to call the doctor	4.73 (± 0.52)
54. Know how to handle equipment (for example, monitors)	4.78 (± 0.53)
53. Know how to give shots, IVs, etc	4.80 (± 0.51)
3. Know what they’re doing	4.87 (± 0.44)

**Table 2:** Mean and standard deviation (± 1SD) for the five lowest‐ranking items

**Item**	**Mean (**± 1**SD)**
62. Help me see that my past experiences are important	3.38 (± 0.91)
21. Ask me what I like to be called	3.37 (± 0.99)
49. Consider my spiritual needs	3.22 (± 1.01)
20. Talk to me about my life outside the hospital	3.09 (± 0.94)
25. Visit me if I move to another hospital unit	2.88 (± 1.06)

### Cronbach’s alpha and MacDonald’s omega scores by subscales

To calculate the mean ± 1SD per subscale, the items were grouped into their respective subscales. Table 3 shows the scores by subscales alongside their reliability coefficients (ω). As expected, the subscale “Existential/phenomenological/spiritual forces” was the lowest-ranking subscale (3.76 ± 0.34), while “Human needs assistance” was the highest-ranking subscale (4.49 ± 0.23). Nevertheless, both Cronbach’s alpha and McDonald’s omega were 0.8 or higher in all subscales. Importantly, Cronbach’s alpha for the overall scale was 0.96, indicating that the instrument shows a high internal consistency, while McDonald’s omega showed high reliability (0, 97).

**Table 3:** Score (Cronbach’s α) and reliability coefficient (McDonald’s ω) by subscales

Rank	Dimension	No. of items	Mean ± 1SD	Cronbach’s alpha	McDonald’s omega
1	Human needs assistance	9	4.49 ± 0.23	0,81	0,85
2	Humanism/faith-hope/sensitivity	16	4.34 ± 0.29	0,92	0,92
3	Teaching/learning	8	4.30 ± 0.97	0,87	0,91
4	Supportive/protective/corrective environment	12	4.09 ± 0.36	0,82	0,87
5	Helping/trust	11	3.93 ± 0.57	0,82	0,87
6	Expression of positive/negative feelings	4	3.77 ± 1.09	0,83	0,85
7	Existential/phenomenological/spiritual forces	3	3.76 ± 0.34	0,80	0,81
	CBA	63	4.10 ± 0.55	0,96	0,97

### Consideration of scale purification

After running the statistical tests, we were dissatisfied with some of the results and deliberated on the need for scale purification [24]. We found that the items correlating less highly with the overall scale, typically those carrying some existential meaning, were not automatically associated by the respondents with nursing care, and some even considered they were not pertinent to nurses’ work.

Additionally, numerous participants informed us that some items were confusing or sounded redundant. This result had already been detected during the linguistic phase of the study (phase 1), when participants often pointed out that some questions were being asked twice, although differently, which they found somewhat tiresome or repetitive (see Table 4).

**Table 4:** Problematic items

Problematic items	
20. Talk to me about my life outside the hospital 21. Ask me what I like to be called 25. Visit me if I move to another hospital unit 49. Consider my spiritual needs 62. Help me see that my past experiences are important	

The decision to perform scale purification for the sake of simplicity required some debate among the listed researchers, as our aim was to have a very high correlation in all of the items. Naturally, this is not the aim of validating an instrument per se. More problematic still were the items that had relatively lower correlations but were meaningful from a theoretical perspective [25].

We thus aimed to combine personal judgement and statistical criteria, as keeping those items could allow changes in perception to be assessed across time. Furthermore, when removing the items in question, the overall Cronbach’s alpha increased only minimally (from 0.960 to 0.963). Therefore, we decided to keep all 63 items, as in the original CBA [19], resulting in the validated version of the CBA questionnaire in Spanish. The final version and the item-by-item translation are provided in the Supplementary material.

### Exploratory factor analysis

Interestingly, EFA showed that while subscales 1, 2 and 5 are conceptually linked (Humanism/Faith-hope/Sensitivity, Helping/trust, Supportive/protective/corrective environment), these were also strongly associated in the dataset. Similarly, subscales 4 and 6 (Teaching/learning, Human needs assistance) and 3 and 7 (Expression of positive/negative feelings, Existential/phenomenological/spiritual forces) formed somewhat 4 separate groupings on their own. This was also highlighted by the parallel analysis, which showed that 5 factors were found. The latter was reassuring in terms of how well structured the CBA tool is. Additionally, EFA enabled us to identify that the highest loadings (L, see Table 5) were item 17 “Really listen to me when I talk” (L = 0.71); item 36 “Ask me what I want to know about my health/illness” (L = 0.70); item 37 “Help me set realistic goals for my health” (L = 0.69); item 06 “Encourage me to believe in myself” (L = 0.69); item 07 “Point out positive things about me and my condition” (L = 0.67); and item 28 “Encourage me to talk about how I feel” (L = 0.67).

**Table 5:** Item loadings (higher than 0.5) per factor

Item Loadings (> 0.5) per Factor					
Item	Factor1	Factor2	Factor3	Factor4	Factor5
5	0.52				
6	0.69				
7	0.67				
8	0.57				
20	0.59				
26	0.61				
28	0.67				
30	0.63				
62	0.55				
63	0.6				
9		0.55			
11		0.52			
12		0.58			
13		0.61			
16		0.58			
17		0.71			
18		0.59			
41			0.56		
43			0.55		
48			0.51		
34				0.62	
35				0.61	
36				0.7	
37				0.69	
38				0.68	
53					0.86
54					0.84
55					0.63
60					0.6

KMO and Bartlett’s sphericity test showed that our data set was able to be factorized. KMO overall was 0.93, while Bartlett’s sphericity test (X_2_ = 11126.8, *p* < 0.05) also suggested that our dataset could be used in EFA. This analysis was done using 5 factors, as shown by the parallel analysis. Table 5 shows the item loadings higher than 0.5 for each factor, while the results for the EFA are shown on Table 6. The first 3 factors explain 30% of observed variability, while adding factors 4 and 5, completed the 45% of variability explanation (see Table 6).

**Table 6:** Exploratory factorial analysis results for each factor

	Factor1	Factor2	Factor3	Factor4	Factor5
SS Loadings	7.15	6.57	5.05	4.98	4.45
Proportion variance	0.11	0.1	0.08	0.08	0.07
Cumulative variance	0.11	0.22	0.3	0.38	0.45

The variability explained after the EFA clearly demonstrates how complex the observed variability becomes following the application of the CBA tool.

### How respondents answered the open‐ended question

Some carefully selected examples of the participants’ responses are shown in Table 7. Additionally, in Phase 1 participants seemed surprised by the items relating to existential/phenomenological/spiritual dimensions. The participants disagreed that these dimensions pertained to nursing care (i.e., “What have nurses become now? Psychologists?”).

**Table 7:** Open-ended responses

Opened-ended responses	
• I would tell nurses that if they say they are going to do 10 things, they should do them. • If I get angry or in a bad mood, keep talking to me. • I would ask nurses to take my family into account when providing information. • I would love it if nurses explained to me what is planned for the day, so there is no uncertainty, and I can organise myself. • That nurses make eye contact, that they respect designated rest schedules and don’t try to wake me up because they must take my blood pressure at 7 in the morning, unless it´s strictly necessary. • It was very unpleasant for me that they sometimes talked as if I were not in the room, for example, when they were helping me to shower, a nurse talked to her colleague about her son’s grades. • Please explain what I need to know about my illness. • I would ask nurses, if they have to do a chest X-ray, for example, that they let me wear pants. • Nurses should understand that, like them, we also have bad days, and that in that situation everything is magnified. • Nurses should avoid pulling faces.	

## Discussion

### Discussion of cultural adaptation and validity of the CBA

The steps taken to ensure accurate cultural adaptation of the Spanish version of the CBA were essential to creating a version tailored to Spanish users, considering the specific features of a region influenced by several languages. Cronbach’s alpha for overall reliability was high (0.96), and all its subscales were 0.8 or higher. The overall Chronbach’s alpha is reassuring as it mirrors that of the Chilean Spanish CBA validated by Ayala and Calvo in 2017 [15], although in our study there was more dispersion across the subscales. Equally, McDonald’s omega showed high reliability.

Research studies conducted in different regions have also validated CBA versions for patients in the USA [26], Saudi Arabia [27] and Jordan [28]. These studies consistently reported overall Cronbach’s alpha values above 0.8, adding cumulative evidence in support of the CBA as a valid instrument to measure nurses’ caring behaviours.

Moreover, a descriptive analysis was conducted to identify the caring behaviours receiving the highest and lowest ranking. As expected, some items showed weaker correlations with the overall scale, and some participants even considered them “irrelevant” or unrelated to nurses’ duties. When we compared our study to that performed by Ayala and Calvo [15] and the original by Cronin and Harrison [19], similarities were found in the results for most of the items. However, differences were found in the item “consider my spiritual needs”, which was rated lower by the Spanish sample. This discrepancy may be related to cultural and contextual factors influencing perceptions and expectations regarding caring behaviours.

### Emergence of a 5-dimensional factorial solution for the CBA scale in the Spanish context

Our study presents evidence for a 5-dimensional factorial solution for the CBA scale in the Spanish healthcare context. The convergence of findings suggests that the identified dimensions capture meaningful variance in the dataset and reflect underlying patterns of caring behaviors within the Spanish healthcare context.

Our findings suggest a strong theoretical coherence among certain dimensions within the CBA (Caring Behavior Assessment) scale, reflecting interconnected clusters of caring behaviors. For instance, subscales 1, 2, and 5 demonstrate conceptual linkage, forming a cohesive first dimension that encompasses ‘Humanism/Faith-hope/Sensitivity, Helping/Trust, and Supportive/Protective/Corrective Environment’. Specifically, our analysis reveals an expanded understanding within the first dimension, encompassing not only the initial three carative factors as in the original version but also incorporating two additional factors. These include the formation of a humanistic-altruistic system of values, the installation of faith-hope, the cultivation of sensitivity to oneself and others, the development of a helping-trust relationship, and the provision for a supportive, protective, and corrective environment. This expanded dimension highlights the interconnectedness of empathy, compassion, trust, and reliability within caregiving relationships, reinforcing the foundational principles outlined in Watson’s Theory of Transpersonal Care [8] and also supported by established theories of patient-centered care [29]. Additionally, this dimension highlights the importance of providing a supportive, protective, and corrective mental, physical, sociocultural, and spiritual environment, aligning closely with Watson’s emphasis on creating conducive environments for healing and growth. By recognizing this evolution in our analysis, we underscore the ongoing refinement and adaptation of theoretical frameworks to specific contexts better capture the complexities of caregiving dynamics and promote holistic patient care.

While subscales 1, 2, and 5 form a single cohesive dimension, subscales 3, 4, 6 and 7, form separate groupings, resulting in a total of five dimensions, each representing specific facets of caring behaviors. The second dimension, ‘Teaching/Learning’, focuses on the educational aspects of caregiving and skills training. This dimension aligns with the principles of transpersonal care, emphasizing the importance of nurturing the growth and development of both caregivers and recipients through shared learning experiences. The third dimension, ‘Human Needs Assistance,’ emphasizes the importance of fulfilling the fundamental needs of people receiving care, reflecting the humanistic approach to caregiving that prioritizes the preservation of dignity and autonomy. The subscale ‘Expression of Positive/Negative Feelings’ captures the acknowledgement and validation of the emotional experiences of patients receiving care, resonating with the empathetic and compassionate aspects of transpersonal care. Lastly, the dimension ‘Existential/Phenomenological/Spiritual Forces’ addresses the existential, phenomenological, and spiritual aspects of caregiving. This dimension emphasizes the interconnectedness of mind, body, and spirit, echoing the holistic perspective of transpersonal care, which acknowledges the spiritual essence and interconnectedness of all beings. This comprehensive framework illuminates the multifaceted nature of caregiving, addressing diverse aspects essential for holistic patient care and well-being.

### Relevant findings and preferences of Spanish individuals

The highest-ranking items among the Spanish participants mainly related to technical and cognitive components, such as competence in clinical procedures and the handling of equipment. Conversely, the lowest-ranking behaviours related to emotional and existential dimensions, such as talking about life outside the hospital, understanding patients’ experiences, and considering spiritual needs. These results may indicate that, within the Spanish context, these components are perceived by patients as less important than technical competencies, thus highlighting their priorities in terms of their care, even though the respondents were not hospitalised. These results suggest that clinical skills and technical competencies play an important role in patients’ perceptions of the quality of nursing care in Spain [30]. This finding is supported by a prior study [31] comparing nursing practice in Spain with that in the UK.

The prioritization of technical competencies over emotional and existential dimensions in nursing care may be explained by people’s prioritizing. Individuals usually prioritize basic needs and gradually move to more complex ones after basic needs are met. The perception of care may follow a similar pattern. The primary focus may thus be on safety and meeting the standard of performance required to guarantee this basic need, with less emphasis on the overall experience of wellbeing and being looked after. This approach also tends to be used in healthcare delivery, where the main focus is usually placed on survival-related outcomes [32]. However, as healthcare evolves toward value-based and person-focused approaches, there is growing awareness of the need to expand services and prioritize broader aspects of care. Expectations may thus be informed by factors such as recovery and quality of life, and become aligned with patients’ priorities, expectations and desire for comprehensive care and enhanced overall quality of life. By understanding this dynamic, healthcare professionals can better navigate the complexities of patient expectations and ensure the delivery of care in accordance with diverse needs and preferences.

However, to ensure comprehensive nursing care aligned with the expectations of individuals in Spain, it is essential to have a deep understanding of their individual needs and priorities. Validation studies conducted for specific populations may shed light on the elements of healthcare that are highly valued and contribute to humanisation. For example, research focusing on transgender populations has shown that being asked about their preferred form of address is highly valued [33] but does not seem to be a priority for the general population in our setting. Similarly, individuals in end-of-life processes place great importance on the ability of nurses and clinicians to show compassion and empathise with their feelings, while these qualities were not prioritised in the participants in our sample [34]. Equally, women going through challenging experiences, such as miscarriage, stressed that a key element of the care they required was being helped to cope with the future and understand their feelings [35].

In a similar vein, another study focused on how the general population perceived the quality of nursing services. The findings of that study revealed that various dimensions of quality, such as psychological, physical, and communication components, were rated at a moderate level, suggesting that there was room for improvement in meeting patients’ expectations [36]. This finding emphasises the importance of tailoring nursing care to specific populations to address the complexity of individual preferences, and highlights the need to focus on the multidimensional aspects of care to enhance the overall quality of nursing activity.

An awareness of contemporary nursing training and the scope of nurses’ work in society could fruitfully contribute to shifting such expectations away from a focus on technical and knowledge-related issues. As stated by López-Verdugo et al. [37], society often relies on misinformation when referring to nursing work, which is also often based on widely disseminated myths and stereotypes. A stereotyped image of nursing work, and of nurses themselves, may well lie beneath the reaction of some of the Spanish participants in our study when asked about the importance of emotional and spiritual needs in nursing care. Participants may not always fully appreciate the importance of integrated care, just as contemporary nursing remains largely unknown in Spain [37]. Therefore, a change in perspective is needed to foster greater appreciation of the profession for more rewarding experiences during periods of health and illness, both for users and for healthcare providers.

Previous research has emphasised human care as a driving force in nursing practice, highlighting that quality care relies on a holistic view of care that extends beyond technical proficiency [38]. Several studies have underscored that human care, which encompasses emotional support, effective communication, and attention to patients’ psychosocial needs, is essential for promoting patient satisfaction and achieving favourable health care outcomes [39].

A drawback of the CBA is its relatively long length, leading to a risk of tiring respondents. This limitation has been acknowledged in previous literature [15]. In addition, during the cultural adaptation phase of the present study, participants reported that some items were somewhat repetitive. To address this concern, future research could focus on validating abbreviated versions of this and other instruments. This approach would allow more streamlined integration of theoretical perspectives into routine assessments in clinical practice. Similarly, exploring the perspectives of specific population groups could provide a more nuanced understanding of their unique expectations regarding healthcare.

As patient-centered care gains recognition as a fundamental aspect of quality healthcare, understanding and measuring caring behaviors become necessary for healthcare organizations and professionals, highlighting the importance of tools like the CBA scale.

## Conclusion

The interplay between theory and practice has gained prominence in nursing care over the past two decades. This dynamic encompasses various dimensions, ranging from abstract concepts like human sensitivity and emotional engagement to more tangible factors such as clinical skills. In this context, the use of tools to assess and translate nursing care into workable data have gained importance in healthcare policy and management. Indeed, such objective data can be useful for decision-makers in higher-level management, as nurses’ work is key to user satisfaction and the transformation of the biomedical paradigm in health care. Adapting and validating instruments can thus contribute to these processes.

Similarly, implementing ‘tooling up’ strategies can be a useful way of rendering nurses’ often invisible work visible, which, in the process, could incentivise a humane approach, which is perceived to have been lost in the evolutionary loop of healthcare in the industrialised world.

To support this endeavour, this article provides a validated version of the CBA for users in Spain. This version remains true to the original CBA but incorporates certain modifications into the Spanish version for respondents’ ease of use. Through a process of translation, cultural adaptation and statistical analysis, this new version has been demonstrated be a valid and culturally-appropriate instrument, which provides reliable, objective, comparable and culturally-sensitive data on patients’ perceptions of the most essential elements of care during hospitalization.

All authors declare that they have no conflicts of interest. The individuals who participated in this study were research participants and were not involved in the design, conduct, or preparation of the manuscript.

## Relevance for clinical practice

The study addressed the problem of the lack of a culturally translated, adapted and culturally validated version of the Caring Behaviors Assessment (CBA) tool in the Spanish context. This was a significant issue as it hindered the collection of objective and culturally sensitive data on essential aspects of care.

The research will have an impact on several groups. First, it will benefit healthcare professionals and providers, policymakers and managers by providing them with a reliable instrument to evaluate and improve patient care. This instrument could enhance their understanding of patient needs and preferences, enabling them to identify areas for improvement and promote person-centered care.

Second, the research could directly benefit the Spanish-speaking population. Through the CBA tool, individuals will be able to ask for care that aligns more closely with their personal values and preferences, thus promoting a shift towards person-centered care.

### Supplementary Information

**Supplementary Material 1.**

## Data Availability

The datasets used and/or analyzsed during the current study are available from the corresponding author on reasonable request.

## Abbreviations

CBA: Caring Behaviors Assessment
EFA: Exploratory Factor Analysis
SD: Standard Deviation
