# Supplementary material for: Cultural adaptation and validation of the caring behaviors assessment tool into Spanish
Source: BMC Nurs. 2024 Apr 10;23:240. doi: 10.1186/s12912-024-01892-2 (PMC11007873; doi:10.1186/s12912-024-01892-2)
Supplement: Supplementary file 1 — Supplementary Material 1. [file 12912_2024_1892_MOESM1_ESM.pdf]

Supplementary file 1. Spanish validation of the original Caring Behaviors Assessment Tool

CARING BEHAVIORS ASSESSMENT TOOL (CBA)

INSTRUMENTO DE EVALUACIÓN DE LOS COMPORTAMIENTOS DE CUIDADO

Reproduced/Translated with permission from Dr. Sherill Cronin

**Original English version CBA scale items (Cronin & Harrison, 1988)**	**Translated and adapted Spanish version**					
Listed below are things nurses might do or say to make you feel cared for and about. Please decide how important each of these would be in making you feel cared for and about.<br>For each item, indicate if it would be of:<br>Much Importance 5 4 3 2 1 Little Importance	*A continuación, se enumeran acciones que los/las enfermeros/as podrían hacer o decir para hacerle sentir que le cuidan. Por favor, indique la importancia que tendría cada una de estas opciones para que sintiera que le cuidan.*<br>*Muy poco importante 1 2 3 4 5 Muy importante*					
1. Treat me as an individual.	*1. Que me traten como una persona única*	1	2	3	4	5
2. Try to see things from my point of view.	*2. Que traten de ver las cosas desde mi punto de vista*	1	2	3	4	5
3. Know what they`re doing.	*3. Que sepan lo que hacen*	1	2	3	4	5
4. Reassure me.	*4. Que me tranquilicen*	1	2	3	4	5
5. Make me feel someone is there if I need them.	*5. Que me hagan sentir que están cerca si los/las necesito*	1	2	3	4	5
6. Encourage me to believe in myself.	*6. Que me animen a creer en mí mismo/a*	1	2	3	4	5
7. Point out positive things about me and my condition.	*7. Que destaquen aspectos positivos sobre mí y mi estado de salud*	1	2	3	4	5
8. Praise my efforts.	*8. Que elogien mis esfuerzos*	1	2	3	4	5
9. Understand me.	*9. Que me comprendan*	1	2	3	4	5

10. Ask me how I like things done.	*10. Que me pregunten cómo me gusta que se hagan las cosas.*	1	2	3	4	5
11. Accept me the way I am.	*11. Que me acepten como soy*	1	2	3	4	5
12. Be sensitive to my feelings and moods.	*12. Que sean comprensivos/as con mis sentimientos y estados de ánimo*	1	2	3	4	5
13. Be kind and considerate.	*13. Que sean amables y considerados/as*	1	2	3	4	5
14. Know when I`ve “had enough“ and act accordingly.	*14. Que se den cuenta de cuando "ya no puedo más" y actúen en consecuencia*	1	2	3	4	5
15. Maintain a calm manner.	*15. Que mantengan la calma*	1	2	3	4	5
16. Treat me with respect.	*16. Que me traten con respeto*	1	2	3	4	5
17. Really listen to me when I talk.	*17. Que realmente me escuchen cuando hablo*	1	2	3	4	5
18. Accept my feelings without judging them.	*18. Que acepten mis sentimientos sin juzgarlos*	1	2	3	4	5
19. Come into my room just to check on me.	*19. Que vengan a mi habitación para saber cómo estoy*	1	2	3	4	5
20. Talk to me about my life outside the hospital.	*20. Que conversen conmigo sobre mi vida fuera del hospital*	1	2	3	4	5
21. Ask me what I like to be called.	*21. Que me pregunten cómo me gusta que me llamen*	1	2	3	4	5
22. Introduce themselves to me.	*22. Que se presenten*	1	2	3	4	5
23. Answer quickly when I call for them.	*23. Que vengan rápido cuando les llamo*	1	2	3	4	5
24. Give me their full attention when with me.	*24. Que me presten toda su atención cuando están conmigo*	1	2	3	4	5
25. Visit me if I move to another hospital unit.	*25. Que me visiten si me trasladan a otra unidad hospitalaria*	1	2	3	4	5
26. Touch me when I need it for comfort.	*26. Que establezcan contacto físico (por ejemplo, me dan la mano) cuando lo necesito para reconfortarme*	1	2	3	4	5
27. Do what they say they Will do.	*27. Que cumplan con lo que dicen que harán*	1	2	3	4	5
28. Encourage me to talk about how I feel.	*28. Que me animen a hablar sobre cómo me siento*	1	2	3	4	5
29. Don`t become upset when I`m angry.	*29. Que no se molesten cuando estoy enfadado/a.*	1	2	3	4	5
30. Help me understand my feelings.	*30. Que me ayuden a entender mis sentimientos*	1	2	3	4	5
31. Don`t give up on me when I´m difficult to get along with.	*31. Que no se rindan cuando es difícil tratar conmigo*	1	2	3	4	5
32. Encourage me to ask questions about my illness and treatment.	*32. Que me animen a hacer preguntas sobre mi enfermedad y tratamiento*	1	2	3	4	5

33. Answer my questions clearly.	*33. Que respondan de manera clara a mis preguntas*	1	2	3	4	5
34. Teach me about my illness	*34. Que me enseñen sobre mi enfermedad*	1	2	3	4	5
35. Ask me questions to be sure I understand.	*35. Que me hagan preguntas para asegurarse que entiendo lo que me dicen*	1	2	3	4	5
36. Ask me what I want to know about my health / illness.	*36. Que me pregunten qué quiero saber sobre mi salud/enfermedad*	1	2	3	4	5
37. Help me set realistic goals for my health.	*37. Que me ayuden a establecer objetivos realistas sobre mi salud*	1	2	3	4	5
38. Help me plan ways to meet those goals.	*38. Que me ayuden a planificar la manera de alcanzar estos objetivos*	1	2	3	4	5
39. Help me plan for my discharge from the hospital.	*39. Que me ayuden a planificar el alta hospitalaria*	1	2	3	4	5
40. Tell me what to expect during the day.	*40. Que me digan qué se espera que suceda durante el día*	1	2	3	4	5
41. Understand when I need to be alone.	*41. Que entiendan cuándo necesito estar solo/a*	1	2	3	4	5
42. Offer things to make me more comfortable.	*42. Que me ofrezcan cosas para hacerme sentir más cómodo/a (p. ej. almohada)*	1	2	3	4	5
43. Leave my room neat after working with me.	*43. Que dejen mi habitación ordenada después de hacer su trabajo*	1	2	3	4	5
44. Explain safety precautions to me and my family.	*44. Que nos expliquen las medidas de precaución a mí y a mi familia*	1	2	3	4	5
45. Give me pain medication when I need it.	*45. Que me den medicación para el dolor cuando lo necesito*	1	2	3	4	5
46. Encourage me to do what I can for myself	*46. Que me animen a hacer por mí mismo aquello que puedo*	1	2	3	4	5
47. Respect my modesty (for example, keeping me covered.)	*47. Que respeten mi intimidad (por ejemplo, manteniéndome tapado/a)*	1	2	3	4	5
48. Check with me before leaving the room to be sure I have everything I need within reach	*48. Que me pregunten antes de salir de la habitación si tengo a mano todo lo que necesito*	1	2	3	4	5
49. Consider my spiritual needs.	*49. Que tengan en cuenta mis necesidades espirituales*	1	2	3	4	5
50. Are gentle with me.	*50. Que sean amables conmigo*	1	2	3	4	5
51. Are cheerful.	*51. Que sean alegres*	1	2	3	4	5
52. Help me with my care until I`m able to do it for myself.	*52. Que me ayuden con mis cuidados hasta que pueda hacerlo por mí mismo/a*	1	2	3	4	5

53. Know how to give shots, IVs, etc.	*53. Que sepan cómo poner inyecciones, vías, etc.*	1	2	3	4	5
54. Know how to handle equipment (for example, monitors).	*54. Que sepan cómo usar los equipos técnicos (por ejemplo, los monitores)*	1	2	3	4	5
55. Give me treatments and medications on time.	*55. Que me den los tratamientos y la medicación con puntualidad*	1	2	3	4	5
56. keep my family informed of progress.	*56. Que mantengan a mi familia informada sobre mi evolución*	1	2	3	4	5
57. Let my family visit as much as possible.	*57. Que permitan que mis familiares me visiten todo lo posible*	1	2	3	4	5
58. Check my condition very closely.	*58. Que vigilen mi estado de salud muy de cerca*	1	2	3	4	5
59. Help me feel like I have some control.	*59. Que me ayuden a sentir que tengo cierto control*	1	2	3	4	5
60. Know when it`s necessary to call the doctor.	*60. Que sepan cuándo hay que llamar al médico*	1	2	3	4	5
61. Seem to know how I feel.	*61. Que muestren que saben cómo me siento*	1	2	3	4	5
62. Help me see that my past experiences are important.	*62. Que me ayuden a ver que mis vivencias pasadas son importantes*	1	2	3	4	5
63. Help me feel good about myself.	*63. Que me ayuden a sentirme bien conmigo mismo/a*	1	2	3	4	5
Is there anything else that nurses could do or say to make you feel cared for and about? If so, what? ________________	*¿Hay algo más que los/las enfermeros/as podrían decir o hacer para hacerle sentir cuidado? En caso afirmativo, especifíquelo:* ________________					
